# The genome of the American groundhog, *Marmota monax*

**DOI:** 10.12688/f1000research.25970.1

**Published:** 2020-09-16

**Authors:** Daniela Puiu, Aleksey Zimin, Alaina Shumate, Yuchen Ge, Jiabin Qiu, Manoj Bhaskaran, Steven L. Salzberg

**Affiliations:** 1 Department of Biomedical Engineering, Johns Hopkins University, Baltimore, MD, 21211, USA; 2 Center for Computational Biology, Whiting School of Engineering, Johns Hopkins University, Baltimore, MD, 21211, USA; 3 Lilly Research Laboratories, Eli Lilly and Company, Indianapolis, IN, USA; 4 Departments of Computer Science and Biostatistics, Johns Hopkins University, Baltimore, MD, USA

**Keywords:** genome assembly, groundhog, woodchuck, genome annotation

## Abstract

We sequenced the genome of the North American groundhog, *Marmota monax*, also known as the woodchuck. Our sequencing strategy included a combination of short, high-quality Illumina reads plus long reads generated by both Pacific Biosciences and Oxford Nanopore instruments. Assembly of the combined data produced a genome of 2.74 Gbp in total length, with an N50 contig size of 1,094,236 bp. To annotate the genome, we mapped the genes from another *M. monax* genome and from the closely related Alpine marmot, *Marmota marmota*, onto our assembly, resulting in 20,559 annotated protein-coding genes and 28,135 transcripts. The genome assembly and annotation are available in GenBank under BioProject PRJNA587092.

## Introduction

Groundhogs ( *Marmota monax*), also known as woodchucks, belong to the same family of ground squirrels as the alpine marmot, *Marmota marmota*. Groundhogs are found throughout the eastern United States and across much of Canada. They are small, ground-dwelling rodents that weigh ~4 kg as adults.

The woodchuck is of interest to biomedical science as a model for Hepatitis B virus (HBV) infection in humans, due to endemic infections of woodchucks with woodchuck hepatitis virus (WHV), which is genetically similar to human HBV and causes a similar course of infection ^1^. Unlike some animal models of hepatocellular carcinoma (HCC) that require immunocompromised animals, woodchucks can develop HCC spontaneously after WHV infection. This propensity makes the woodchuck a promising model of HBV-induced hepatocellular carcinoma in humans. This in turn motivated our efforts to sequence, assemble, and annotate its genome.

## DNA isolation

DNA was collected from a healthy, wild-caught adult male woodchuck (WC2) captured in 2016 near Ithaca, New York by Northeastern Wildlife, Inc. The gDNA was isolated from the left medial lobe of the liver from animal WC2. All DNA used for sequencing came from the same animal.

## Results

We generated 3.17 billion paired, 150-bp Illumina reads, for a total of 951 Gbp or approximately 390X genome coverage. We generated 32 million reads using Pacific Biosciences sequencing technology, of which 2.59 million were at least 10,000 bp long. The long PacBio reads contained 42.0 Gbp and had an N50 length of 16,554 bp. We also generated 6.4 million Oxford Nanopore (ONT) reads, of which 1.57 million were at least 10,000 bp long. The long ONT reads totaled 22.2 Gbp and had an N50 length of 13,815 bp. We then assembled the Illumina reads, the PacBio 10Kb+ reads, and the ONT 10Kb+ reads using MaSuRCA v3.2.7 ^2^.

The resulting assembly, Woodchuck_1.0, consists of 8,860 contigs containing 2,737,034,741 bp, with an N50 contig size of 1,094,236. We compared our assembly to a recently published assembly of another woodchuck from the same species, GenBank accession GCA_901343595.1 ^3^. That assembly (MONAX5) was generated entirely from Illumina reads, and it has a total length of 2,552,052,516 bp in 48,534 scaffolds, with a scaffold N50 of 892 kb and a contig N50 of 74,495 bp. The earlier assembly is thus ~185 Mbp shorter than Woodchuck_1.0.

We aligned all contigs and scaffolds between the two assemblies, and found that 3791 scaffolds in MONAX5 were contained within longer contigs in Woodchuck_1.0, with an average identity of 99.24%. In contrast, only 84 contigs from Woodchuck_1.0 were contained in MONAX5 scaffolds, consistent with the much larger contig sizes in our assembly.

We mapped the annotation from MONAX5 to Woodchuck_1.0 using Liftoff ^4^. To assign functions to the mapped transcripts, we aligned them to transcripts annotated in the Alpine marmot ( *M. marmota*, GenBank accession GCA_001458135.1 ^5^. This yielded 20,559 protein-coding genes with 28,135 transcripts (including alternative splice variants). 10,664 of the genes were assigned functions based on near-identical matches with the Alpine marmot annotation, and the rest were labeled as hypothetical proteins. The average transcript contains 7.9 exons.

## Data availability

Data from *Marmota monax* is available at NCBI under BioProject PRJNA587092, including the assembly with annotation at GenBank accession WJEC00000000, and the read data in the Sequence Read Archive under the same BioProject. The assembly and annotation are also available at ftp://ftp.ccb.jhu.edu/pub/data/Groundhog.
