# Supplementary material for: The genome of the American groundhog, Marmota monax
Source: F1000Res. 2020 Sep 16;9:1137. [Version 1] doi: 10.12688/f1000research.25970.1 (PMC7682491; doi:10.12688/f1000research.25970.1)
Supplement: Peer review history

# Peer review: Reviewer response for version 1

**DOI:** 10.5256/f1000research.28660.r74309

**Published:** 2020-11-20

**Authors:** Mihai Pop

**Affiliations:** 1 Center for Bioinformatics and Computational Biology, Institute for Advanced Computer Sciences, University of Maryland, College Park, MD, USA

This manuscript describes the sequencing, assembly, and annotation of the groundhog genome. Overall the manuscript is clearly written and provides a good level of detail about the experimental setup and validation.

 It would be beneficial for the readers if full details of the analytical pipeline (including specific parameters used and quality control/trimming steps) were made available either as supplementary material or through some other online platform (e.g. the authors' FTP server).

 Given the substantial investment in the sequencing of the genome I am surprised that the project did not also generate RNA-seq data from the same animal. Since the rationale for this project is to support the use of the groundhog as a model organism for HCC, it's likely that animals are sacrificed during research making available a range of tissues from which a fairly comprehensive picture of the transcriptome can be gleaned.

Are sufficient details of methods and materials provided to allow replication by others?

Partly

Is the rationale for creating the dataset(s) clearly described?

Yes

Are the datasets clearly presented in a useable and accessible format?

Yes

Are the protocols appropriate and is the work technically sound?

Yes

Reviewer Expertise:

Genomics, genome assembly.

I confirm that I have read this submission and believe that I have an appropriate level of expertise to confirm that it is of an acceptable scientific standard.

# Peer review: Reviewer response for version 1

**DOI:** 10.5256/f1000research.28660.r71457

**Published:** 2020-11-09

**Authors:** Markus Ralser, Toni Gossmann

**Affiliations:** 1 Molecular Biology of Metabolism Laboratory, The Francis Crick Institute, London, UK; 2 Bielefeld University, Bielefeld, Germany

The manuscript by Puiu *et al*. announces an improved, better annotated, and more complete genome of the American groundhog ( *M. monax*), a ground squirrel species. The authors justify the importance of the genome as a model for Hepatitis infections, which sounds plausible. Obviously, such a genome is also important for other disciplines like evolutionary genetics, and as Marmots are exquisitely niche adapted species, a better understanding of their biology helps to understand problems of global importance, i.e. climate change, which warrants highlighting.

 The paper is well written and technically of high quality. I have some questions concerning the relationship to the annotated *M. monax* genome deposited in GenBank (MONAX5), which the authors use as comparison of their genome quality. The authors conclude that their genome is more complete than the deposited genome. As the better quality is a key aspect of this submission, the manuscript would profit to disentangle the relative contribution of technical differences (e.g. due to novel sequencing technologies and assembly strategies) and biological differences (e.g. species variation) or what the relative contribution of each of the parts are. 

 Also, the improved genome annotation builds upon comparative mapping of the Alpine marmot and the deposited *M. monax* genome - meaning that “novel” regions in the new assembly are not covered in a similar quality (potential annotation bias). Also the genome assemblies’ DNA stems from the same individual, which means that the authors may want to include information on heterozygosity to conclude how representative the chosen individuals’ genome is of the species.

 Minor remark: I do not think that a 4 kg rodent would be referred to as “small”; that perception may come from a comparison to some other marmots, but it may feel different in the context of other rodents.

Are sufficient details of methods and materials provided to allow replication by others?

Yes

Is the rationale for creating the dataset(s) clearly described?

Yes

Are the datasets clearly presented in a useable and accessible format?

Yes

Are the protocols appropriate and is the work technically sound?

Yes

Reviewer Expertise:

Metabolism

We confirm that we have read this submission and believe that we have an appropriate level of expertise to confirm that it is of an acceptable scientific standard.
